# Supplementary material for: PeakDecoder enables machine learning-based metabolite annotation and accurate profiling in multidimensional mass spectrometry measurements
Source: Nat Commun. 2023 Apr 28;14:2461. doi: 10.1038/s41467-023-37031-9 (PMC10147702; doi:10.1038/s41467-023-37031-9)
Supplement: Supplementary file 1 — Supplementary information [file 41467_2023_37031_MOESM1_ESM.pdf]

PeakDecoder enables machine learning-based metabolite annotation and accurate profiling in multidimensional mass spectrometry measurements

Aivett Bilbao[1,2*], Nathalie Munoz[1,2], Joonhoon Kim[1,2], Daniel J Orton[1], Yuqian Gao[1,2], Kunal Poorey[3], Kyle R. Pomraning[1,2], Karl Weitz[1], Meagan Burnet[1], Carrie D. Nicora[1], Rosemarie Wilton[4,2], Shuang Deng[1,2], Ziyu Dai[1,2], Ethan Oksen[5], Aaron Gee[6], Rick A. Fasani[6], Anya Tsalenko[6], Deepti Tanjore[5,2], James Gardner[5,2], Richard D. Smith[1], Joshua K. Michener[7,2], John M. Gladden[3,2], Erin S. Baker[8], Christopher J. Petzold[5,2], Young-Mo Kim[1,2], Alex Apffel[6], Jon K. Magnuson[1,2] and Kristin E. Burnum-Johnson[1,2*]

[1] Pacific Northwest National Laboratory, Richland, WA, USA
[2] US Department of Energy, Agile BioFoundry, Emeryville, CA, USA
[3] Sandia National Laboratory, Livermore, CA, USA
[4] Argonne National Laboratory, Lemont, IL, USA
[5] Lawrence Berkeley National Laboratory, Berkeley, CA, USA
[6] Agilent Research Laboratories, Agilent Technologies, Santa Clara, CA, USA
[7] Oak Ridge National Laboratory, Oak Ridge, TN, USA
[8] Department of Chemistry, University of North Carolina, Chapel Hill, NC, USA

These authors contributed equally: Aivett Bilbao, Nathalie Munoz, and Joonhoon Kim.

* Correspondence:
Aivett.Bilbao@pnnl.gov and Kristin.Burnum-Johnson@pnnl.gov

Supplementary information

Compound_I	Method_HILIC_mi	Method_HILIC_plus	Method_RP_mi	Method_RP_plus
1005	86.33	5.62	77.75	9.04
10267	94.67	44.56	15.31	27.93
1060	62.27	0.00	0.00	8.60
10690	93.31	1.90	0.00	9.56
1081	56.16	0.00	11.08	66.65
1110	88.15	0.00	6.03	8.60
119	96.37	96.70	6.03	6.99
1195	8.38	0.00	4.32	2.50
1198	92.77	1.90	93.81	46.85
122357	48.88	13.43	0.02	0.07
124886	98.73	97.60	95.91	37.34
134490	97.98	85.17	6.03	6.99
165007	55.34	44.56	0.46	24.50
289	87.20	0.00	1.62	0.32
3035456	93.31	1.90	0.00	9.56
311	93.02	6.05	93.81	67.47
33032	97.18	99.80	0.10	6.99
439183	90.92	17.57	0.00	0.07
439184	41.21	44.56	0.18	0.07
439230	79.67	6.05	93.81	46.85
439284	94.67	44.56	15.31	27.93
439400	79.67	29.10	93.81	41.57
439418	79.67	6.05	93.81	41.57
440101	94.67	44.56	15.31	27.93
440641	94.67	44.56	15.31	27.93
444212	90.60	0.69	90.88	92.03
444493	97.52	99.90	24.24	44.81
444972	84.81	20.21	79.87	90.52
445127	94.68	99.90	61.22	52.71
445713	4.30	0.00	5.35	2.50
445995	4.30	0.00	5.35	2.50
447277	4.30	0.00	5.35	2.50
51	78.73	6.05	0.10	8.60
525	91.78	0.00	6.03	9.56
5280518	84.81	20.21	79.87	90.52
5356793	84.81	20.21	79.87	90.52
5793	100.00	1.90	0.00	0.00
5950	34.30	23.71	0.00	8.60
5960	65.32	48.26	6.03	8.60
5961	100.00	100.00	2.30	4.70
6029	99.50	34.66	96.96	96.45
6030	99.50	40.72	96.96	24.49
60961	99.80	100.00	49.72	80.95
612	21.04	0.00	0.00	9.56
6140	100.00	99.59	80.64	99.91
6251	100.00	1.90	0.00	0.00
6288	98.00	97.04	6.03	9.56
6305	99.90	96.80	95.96	83.72
643757	90.60	0.69	90.88	92.03
644066	94.68	99.90	54.37	44.81
647	10.42	0.00	5.35	2.50
6508	94.73	1.90	21.80	0.13
65533	94.67	44.56	15.31	27.93
668	32.93	1.92	0.34	0.07
68152	70.75	0.00	0.00	9.56
6912	100.00	1.90	0.00	0.00
72	85.39	0.00	100.00	40.37
729	77.03	17.57	0.03	0.07
7427	100.00	1.90	0.00	0.00
811	90.60	0.69	90.50	77.23
8629	99.67	34.66	96.96	24.49
91493	93.31	44.56	0.00	0.07
92133	94.68	99.90	54.37	44.81
92153	97.52	99.90	24.24	44.81
94154	100.00	1.90	0.00	0.00

Supplementary Figure 1. Probabilities calculated by our chromatographic prediction tool for the initial 64 metabolites of interest. The first column indicates PubChem IDs. Four methods were evaluated: HILIC negative ESI, HILIC positive ESI, RP negative ESI and RP positive ESI. Green colors indicate higher probabilities and red colors indicate lower probabilities. HILIC negative ESI maximized the number or metabolites detected, with only 14 molecules with <70% probability and only 10 molecules with <50% probability. Source data are provided as a Source Data file.

Supplementary Table 1. Metabolites of interest and their RT and CCS (deprotonated ion) determined from the standards.

Molecule	PubChemID	Neutral Formula	Exact Mass	Retention Time	Collisional Cross Section [M-H]
01_malic acid	525	C4H6O5	134.021523	2.2	116.25
03_cis-aconitic acid	643757	C6H6O6	174.016438	2	124.63
04_trans-aconitic acid	444212	C6H6O6	174.016438	3.5	126.92
05_isocitric acid	1198	C6H8O7	192.027003	3.5	127.43
06_citric acid	311	C6H8O7	192.027003	4.9	127.49
07_succinic acid	1110	C4H6O4	118.026609	1.8	116.56
08_alpha-ketoglutaric acid (aKG)	51	C5H6O5	146.021523	1.8	121.43
09_lactic acid	612	C3H6O3	90.0316941	1.2	113
10_3-hydroxypropanoic acid (3HP)	68152	C3H6O3	90.0316941	1.3	113.8
11_fumaric acid	444972	C4H4O4	116.010959	2.2	117.64
12_pyruvic acid	1060	C3H4O3	88.016044	0.9	111.67
13_aspartic acid	5960	C4H7NO4	133.037508	2.2	119.13
14_citramalic acid	1081	C5H8O5	148.037173	1.8	122.18
15_glyceraldehyde-3-P (G3P)	729	C3H7O6P	169.998025	2.4	124.95
16_phosphoenolpyruvate (PEP)	1005	C3H5O6P	167.982375	3.3	121.84
17_glucose 6-phosphate (G6P)	439284	C6H13O9P	260.029719	3.6	147.27
18_fructose 6-phosphate (F6P)	440641	C6H13O9P	260.029719	3.2	144.3
19_fructose 1,6-diphosphate (F16DP)	10267	C6H14O12P2	339.99605	5	155
20_6-phosphogluconic acid (6PG)	91493	C6H13O10P	276.024634	4.8	144.08
21_3-phosphoglyceric acid (3PG)	439183	C3H7O7P	185.99294	4.8	125.05
22_quinic acid	6508	C7H12O6	192.063388	1.5	134.75
23_protocatechuic acid	72	C7H6O4	154.026609	1.2	123.03
24_acetoacetyl-CoA	92153	C25H40N7O18P3S	851.13634	3.3	254.51
25_HMG-CoA	445127	C27H44N7O20P3S	911.157469	4.8	261.39
26_mevalonic acid	439230	C6H12O4	148.073559	1.1	128.97
27_mevalonate-5-phosphate (mev-5P)	439400	C6H13O7P	228.03989	3	138.11
28_mevalonate 5-diphosphate (mev-5PP)	439418	C6H14O10P2	308.006221	4.8	151.89
29_isopentenyl diphosphate (IPP)	1195	C5H12O7P2	246.005827	2.1	144.98
31_geranyl diphopshate (GPP)	445995	C10H20O7P2	314.068427	1.6	167.33
32_farnesyl diphosphate (FPP)	445713	C15H28O7P2	382.131027	1.2	186.96
33_geranygeranyl diphosphate (GGPP)	447277	C20H36O7P2	450.193628	1.1	205.78
34_itaconic acid	811	C5H6O4	130.026609	1.4	118.68
35_gluconic acid	10690	C6H12O7	196.058303	1.7	132.86
36_2-ketogluconic acid	3035456	C6H10O7	194.042653	1.6	131.66
37_ribulose 5-phosphate	439184	C5H11O8P	230.019154	2.5	138.7
38_ribose 5-phosphate	440101	C5H11O8P	230.019154	2.7	139.33

39_glutamic acid	33032	C5H9NO4	147.053158	2	124.39
40_alanine	5950	C3H7NO2	89.0476785	1.6	116.55
41_UDP-glucose	8629	C15H24N2O17 P2	566.055022	3.1	207.17
42_glucose 1-phosphate (G1P)	65533	C6H13O9P	260.029719	3.4	148.5
43_cis,cis-muconic acid	5280518	C6H6O4	142.026609	1.6	121.82
44_trans, trans-muconic acid	5356793	C6H6O4	142.026609	1.9	130.52
45_dihydroxyacetone phosphate (DHAP)	668	C3H7O6P	169.998025	2.4	124.95
46_glutamine	5961	C5H10N2O3	146.069142	1.8	127.59
47_xylitol	6912	C5H12O5	152.068474	1.2	125.27
48_trehalose	7427	C12H22O11	342.116212	1.8	169.82
49_phenylalanine	6140	C9H11NO2	165.078979	1.2	139.88
50_arabitol	94154	C5H12O5	152.068474	1.2	125.27
51_catechol	289	C6H6O2	110.03678	0.8	115.52
52_glucose	5793	C6H12O6	180.063388	1.3	141.57
53_erythrose 4-phosphate (E4P)	122357	C4H9O7P	200.00859	3.7	148.82
54_sedoheptulose 7-phosphate (S7P)	165007	C7H15O10P	290.040284	3.3	150.44
55_tryptophan	6305	C11H12N2O2	204.089878	1.2	151.79
56_glutathione reduced	124886	C10H17N3O6S	307.083806	2.3	162.37
57_mannitol	6251	C6H14O6	182.079038	1.3	131.99
58_uridine	6029	C9H12N2O6	244.069536	1	151.12
59_adenosine	60961	C10H13N5O4	267.096754	1	157.91
60_acetyl-CoA	444493	C23H38N7O17 P3S	809.125775	3.4	248.76
61_malonyl-CoA	644066	C24H38N7O19 P3S	853.115604	4.9	248.74
62_succinyl-CoA	92133	C25H40N7O19 P3S	867.131254	4.8	252.78
63_uridine monophosphate	6030	C9H13N2O9P	324.035867	2.7	161.47
64_L-threonine	6288	C4H9NO3	119.058243	1.6	120.69
65_4-aminobutyric acid (GABA)	119	C4H9NO2	103.063329	1.7	122.64
66_2,4-diaminobutanoic acid	134490	C4H10N2O2	118.074228	3.3	124.07

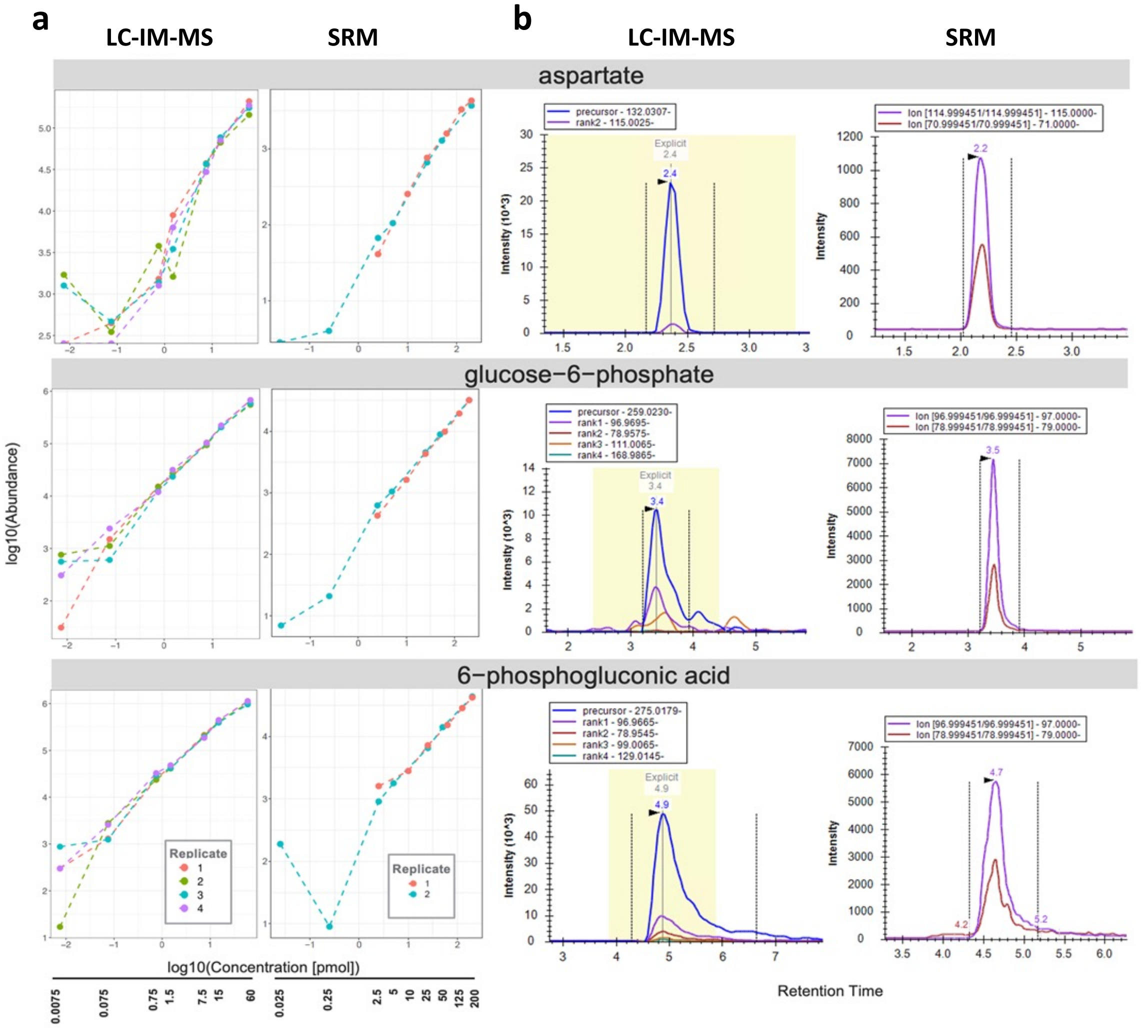

Supplementary Figure 2. Dilution experiments of selected metabolites illustrating the increased sensitivity of LC-IM-MS over SRM. a Calibration curves show linearity for concentrations to as low as 0.075 pmol by IM and 2.5 pmol by SRM (no peak was detected for lower concentrations). The sum of more fragments plus the signal from the intact precursor in DIA can increase the sensitivity compared to the fewer fragments used in the SRM method (typically 2-3). A logarithmic scale was employed to better visualize the full dynamic range. Technical replicates are shown in different colors, exemplifying the good reproducibility of the UHPLC system. For aspartate, a lower ionization efficiency was observed. Source data are provided as a Source Data file. **b** Chromatogram of precursor ions and corresponding transitions of metabolites acquired by IM and SRM.

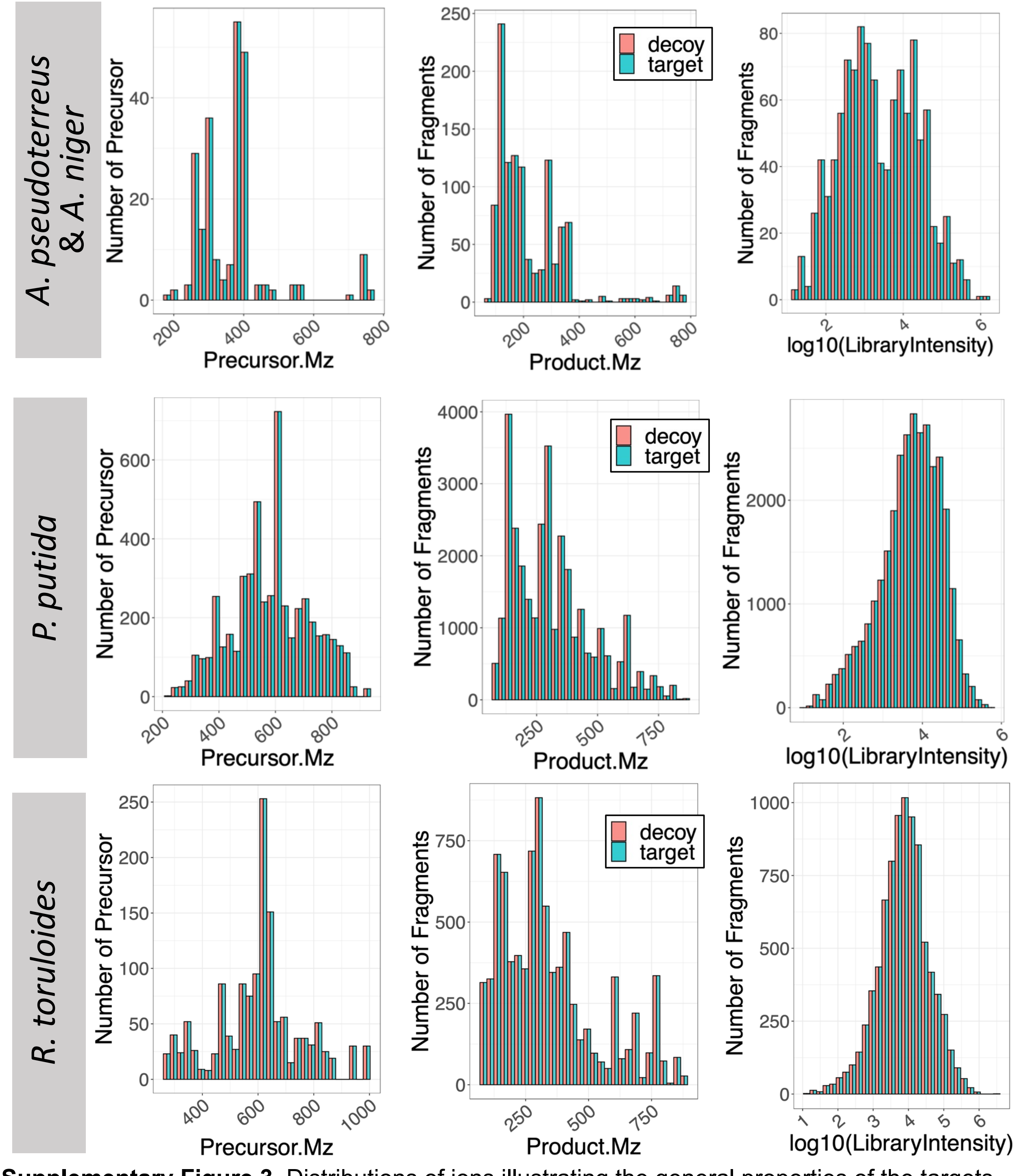

Supplementary Figure 3. Distributions of ions illustrating the general properties of the targets and decoys generated as training sets in all microbial samples.

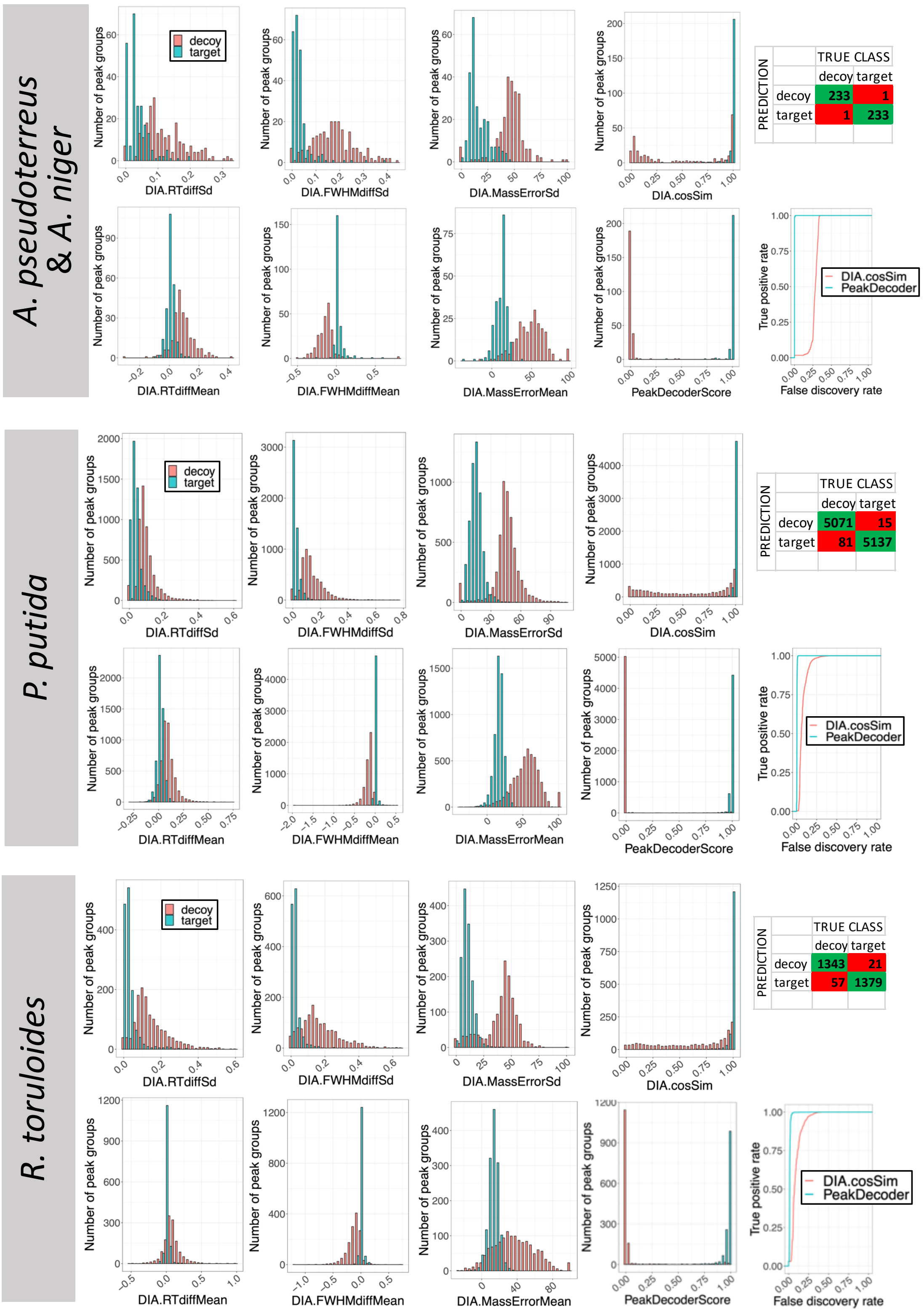

Supplementary Figure 4. Comparison of individual scores and combined PeakDecoder score for training in all microbial samples. Confusion matrixes and true positive vs. false discovery rates are also included. Source data are provided as a Source Data file.

**Sigma**	0.1	0.1	0.5	0.9	0.9	0.5	0.9
**Amplitude**	100	300	300	300	3000	15000	30000
**Accuracy**	99.34	99.46	98.86	99.48	99.65	99.06	96.66
**FDR (%)**	3	2	1	2	1	4	0
**Score**	0.99	0.99	0.96	0.99	0.99	0.97	-

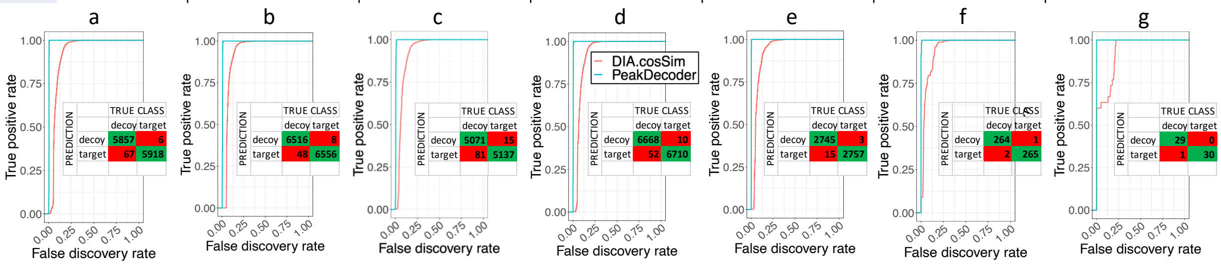

Supplementary Figure 5. Evaluation of the impact of deconvolution parameters in PeakDecoder training. The main deconvolution parameter in MS-DIAL is the sigma window: a higher value (0.7-1.0) will decrease the number of resolved peaks and a lower value (0.1-0.3) may result in many noisy chromatographic peaks. The Sigma value does not directly affect the training performance because PeakDecoder does not use the deconvolution results directly to train the model, it uses that information to generate a preliminary training set as coordinates and performs targeted data extraction with Skyline for both targets and decoys, then it uses the XIC metrics to apply filtering for high-quality fragments to keep high-quality peak-groups as targets and their corresponding decoys. By re-extracting the signals and using only high-quality peak-groups, the potential effect of poorly deconvoluted peak-groups is minimized (panels a-e). Amplitude refers to the minimum precursor intensity used for peak detection. A very large amplitude will result in a drastic filtering of peak-groups and thus a very small training set, which will negatively impact the performance and produce a poor classifier (panels f-g). Accuracy is the average 10-fold cross-validation. If the classifier results in a close-to-perfect accuracy (>99), the minimum non-zero FDR that can be estimated will be affected because the number of false positives is too small. A sigma with a medium value of 0.5 resulted in a good tradeoff to generate sufficient training data for annotations at 1% estimated FDR. Bottom row: confusion matrixes and true positive vs. false discovery rates. Results from the *P. putida* samples (n=22). Source data are provided as a Source Data file.

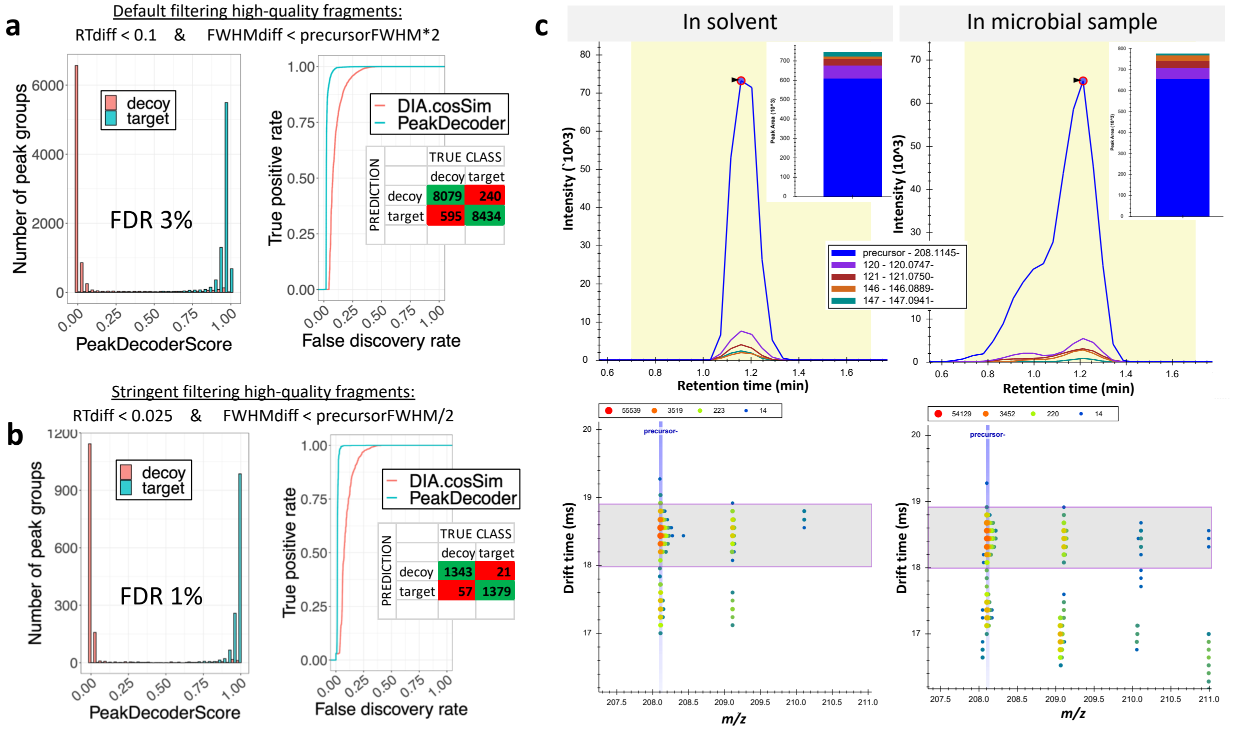

Supplementary Figure 6. Evaluation of PeakDecoder in the *R. toruloides* samples (highest complexity). a PeakDecoder training performance using default filtering of high-quality fragments and 8674 targets/decoys. Source data are provided as a Source Data file. **b** PeakDecoder training performance using stringent filtering of high-quality fragments and 1400 targets/decoys. The higher sample complexity increased the likelihood of low-quality fragments in the deconvoluted peak-groups, therefore, a stringent filtering was necessary to obtain a the minimum non-zero estimated FDR of 1% and a monotonic increasing distribution of PeakDecoder scores for targets (green bars). Source data are provided as a Source Data file. **c** Example from a deuterated standard (tryptophan d5) spiked in solvents and in a microbial sample matrix (*R. toruloides*) to a final concentration of 50 uM. Despite the interfering peak at earlier RT in the complex microbial background, the internal standard was annotated with a PeakDecoder score of 0.9816 (20V CE, model b). Chromatograms show comparable relative abundances in the standard and the microbial sample confirming the correct metabolite annotation based on fragmentation pattern and RT. The IM frame at the LC apex shows the filtering window corresponding to the expected CCS and highlights the precursor with multiple isotopic peaks. Note: none of the library metabolites or the internal standard were used for training.

Supplementary Table 2. Scores and annotation confidence level (best replicate per metabolite) in the *A. pseudoterreus* & *A. niger* dataset.

PrecursorName	Mass.Error.PPM	RetentionTime.Error	CCS.Error	PeakDecoderScore	ConfidenceDescription
01_malic acid	10.6	0.01	-0.1	0.99834986	RT-CCS-DIA
03_cis-aconitic acid	14.4	0.05	0.16	0.998342611	RT-CCS-DIA
04_trans-aconitic acid	9	0.18	-0.07	0.998793441	RT-CCS-DIA
05_isocitric acid	13.7	-0.08	-0.35	0.999301091	RT-CCS-DIA
06_citric acid	11.7	0.21	0.39	0.998818685	RT-CCS-DIA
07_succinic acid	5.3	0	-0.08	0.998260199	RT-CCS-DIA
08_alpha-ketoglutaric acid (aKG)	-7.8	0.06	-0.02	NA	RT-CCS
09_lactic acid	6.7	0.05	-0.47	NA	RT-CCS
10_3-hydroxypropanoic acid (3HP)	4.3	-0.03	-0.23	NA	RT-CCS
11_fumaric acid	3.8	0.04	0.05	0.998937118	RT-CCS-DIA
12_pyruvic acid	-4.9	0.09	0.04	NA	RT-CCS
13_aspartic acid	-3.5	0.03	-0.73	0.998585552	RT-CCS-DIA
14_citramalic acid	6.1	0.29	0.09	0.999334492	RT-CCS-DIA
17_glucose 6-phosphate (G6P)	6.8	-0.29	0.76	0.905403872	RT-CCS-DIA
18_fructose 6-phosphate (F6P)	6.1	0.07	0.8	0.997771751	RT-CCS-DIA
21_3-phosphoglyceric acid (3PG)	-2.3	-0.01	0.17	NA	RT-CCS
35_gluconic acid	13.6	0.04	-0.04	0.998401432	RT-CCS-DIA
39_glutamic acid	4.7	0.09	0	0.998122191	RT-CCS-DIA
40_alanine	7.9	-0.02	0.3	NA	RT-CCS
41_UDP-glucose	10.7	0.16	0.13	0.99892641	RT-CCS-DIA
42_glucose 1-phosphate (G1P)	10.1	-0.07	0.59	0.998776262	RT-CCS-DIA
43_cis,cis-muconic acid	-0.7	-0.02	-0.11	NA	RT-CCS
46_glutamine	14.3	-0.07	-0.06	0.998405725	RT-CCS-DIA
47_xylitol	6.6	0.03	-0.16	0.998860748	RT-CCS-DIA
48_trehalose	13.9	0.05	0.05	0.998170695	RT-CCS-DIA
49_phenylalanine	16.8	-0.01	-0.33	0.998469135	RT-CCS-DIA
50_arabitol	11.5	0.03	-0.16	0.998615518	RT-CCS-DIA
52_glucose	12.1	0.05	0	0.998245972	RT-CCS-DIA
55_tryptophan	9.3	-0.03	-0.28	0.998485906	RT-CCS-DIA
56_glutathione reduced	5.4	-0.14	-0.3	0.998423544	RT-CCS-DIA
57_mannitol	14.3	0.03	0.34	0.99629368	RT-CCS-DIA
58_uridine	12.6	0.06	-0.19	0.998984176	RT-CCS-DIA
59_adenosine	15	0.03	0.06	0.998808412	RT-CCS-DIA
64_L-threonine	5.3	0.01	-0.14	0.999219149	RT-CCS-DIA
65_4-aminobutyric acid (GABA)	1.9	0.03	-0.27	NA	RT-CCS
66_2,4-diaminobutanoic acid	-4.8	-0.03	-0.28	NA	RT-CCS
p01_Aspartate semialdehyde	12.7	0.05	0.05	NA	RT-CCS
p02_Succinate semialdehyde	3.6	0.16	0.01	NA	RT-CCS

Supplementary Table 3. Scores and annotation confidence level (best replicate per metabolite) in the *P. putida* dataset.

PrecursorName	Mass.Error.PPM	RetentionTime.Error	CCS.Error	PeakDecoderScore	ConfidenceDescription
01_malic acid	14.4	-0.12	-0.37	0.976098219	RT-CCS-DIA
05_isocitric acid	12.5	-0.2	0.19	0.988129706	RT-CCS-DIA
06_citric acid	12.5	-0.22	0.14	0.987143569	RT-CCS-DIA
07_succinic acid	13.6	-0.03	-0.35	0.991127708	RT-CCS-DIA
08_alpha-ketoglutaric acid (aKG)	7.7	-0.02	-0.07	NA	RT-CCS
09_lactic acid	10.6	-0.03	0.2	NA	RT-CCS
11_fumaric acid	13	-0.07	0.67	NA	RT-CCS
12_pyruvic acid	13.5	0.01	-0.25	NA	RT-CCS
13_aspartic acid	17.8	-0.07	-0.13	0.994357592	RT-CCS-DIA
15_glyceraldehyde-3-P (G3P)	13.6	-0.07	-0.19	NA	RT-CCS
16_phosphoenolpyruvate (PEP)	14.9	-0.15	-0.03	NA	RT-CCS
17_glucose 6-phosphate (G6P)	16.4	-0.1	-0.13	0.986600036	RT-CCS-DIA
18_fructose 6-phosphate (F6P)	14.5	-0.29	-0.11	0.989192508	RT-CCS-DIA
19_fructose 1,6-diphosphate (F16DP)	9.7	-0.05	0.67	0.990549976	RT-CCS-DIA
20_6-phosphogluconic acid (6PG)	11.6	-0.29	-0.24	0.982117843	RT-CCS-DIA
21_3-phosphoglyceric acid (3PG)	16.1	-0.05	-0.09	0.9892508	RT-CCS-DIA
23_protocatechuic acid	-13	0.03	-0.35	0.989469824	RT-CCS-DIA
29_isopentenyl diphosphate (IPP)	13.1	-0.01	0.37	NA	RT-CCS
31_geranyl diphopshate (GPP)	2	0.18	0.51	NA	RT-CCS
35_gluconic acid	8.8	0.04	-0.28	0.989226747	RT-CCS-DIA
36_2-ketogluconic acid	3.1	0.1	-0.06	NA	RT-CCS
37_ribulose 5-phosphate	15	0.14	0.31	0.984610159	RT-CCS-DIA
39_glutamic acid	14.8	0.05	-0.26	0.988554067	RT-CCS-DIA
41_UDP-glucose	16.3	-0.15	0.4	0.993102325	RT-CCS-DIA
43_cis,cis-muconic acid	1.2	0.04	-0.38	NA	RT-CCS
45_dihydroxyacetone phosphate (DHAP)	10.4	-0.03	-0.19	0.994707603	RT-CCS-DIA
46_glutamine	10.3	-0.02	-0.32	NA	RT-CCS
48_trehalose	10.9	0.09	-0.16	0.990281595	RT-CCS-DIA
49_phenylalanine	9.8	0.03	0.15	0.990271018	RT-CCS-DIA
53_erythrose 4-phosphate (E4P)	11	-0.2	-0.4	0.991334998	RT-CCS-DIA
54_sedoheptulose 7-phosphate (S7P)	14.8	-0.15	-0.14	0.988104432	RT-CCS-DIA
55_tryptophan	15.3	0.02	0.15	0.98639454	RT-CCS-DIA
57_mannitol	14.8	0.01	0.1	0.989269973	RT-CCS-DIA
60_acetyl-CoA	10.7	0.02	-0.23	0.993007312	RT-CCS-DIA
63_uridine monophosphate	13.2	-0.3	-0.18	0.992546856	RT-CCS-DIA
64_L-threonine	2.5	0.29	0.45	0.987656688	RT-CCS-DIA
66_2,4-diaminobutanoic acid	4.7	0.08	0.3	NA	RT-CCS

Supplementary Table 4. Scores and annotation confidence level (best replicate per metabolite) in the *R. toruloides* dataset.

PrecursorName	Mass.Error.PPM	RetentionTime.Error	CCS.Error	PeakDecoderScore	ConfidenceDescription
01_malic acid	4.9	-0.21	0.04	0.987001016	RT-CCS-DIA
05_isocitric acid	-2.7	-0.04	0.36	0.972267343	RT-CCS-DIA
07_succinic acid	-2.5	-0.12	0.07	0.986298499	RT-CCS-DIA
08_alpha-ketoglutaric acid (aKG)	-8.5	-0.06	0.02	NA	RT-CCS
11_fumaric acid	4.8	-0.2	0.19	0.986580804	RT-CCS-DIA
14_citramalic acid	9.9	0.15	0.12	0.985925719	RT-CCS-DIA
17_glucose 6-phosphate (G6P)	-6.2	-0.26	-0.21	NA	RT-CCS
20_6-phosphogluconic acid (6PG)	-6.8	0.01	-0.3	0.984190336	RT-CCS-DIA
25_HMG-CoA	-12.8	-0.26	-0.57	0.961747569	RT-CCS-DIA
26_mevalonic acid	7.4	-0.25	-0.5	NA	RT-CCS
27_mevalonate-5-phosphate (mev-5P)	-7.4	0.03	0.1	NA	RT-CCS
28_mevalonate 5-diphosphate (mev-5PP)	-15.8	0.02	-0.21	NA	RT-CCS
29_isopentenyl diphosphate (IPP) *[M+Na-2H]	11.3	0.11	0.05	NA	RT-CCS
31_geranyl diphopshate (GPP) *[M-H]	15.6	0.05	0.27	NA	RT-CCS
35_gluconic acid	-4.9	0.07	-0.17	0.981816613	RT-CCS-DIA
36_2-ketogluconic acid	-9.3	0.27	0.06	0.966309649	RT-CCS-DIA
38_ribose 5-phosphate	-8.6	-0.27	-0.12	0.980412243	RT-CCS-DIA
39_glutamic acid	0.6	0.04	0	0.9829748	RT-CCS-DIA
40_alanine	-3	-0.3	-0.4	NA	RT-CCS
41_UDP-glucose	-1.8	-0.11	0.31	0.982326672	RT-CCS-DIA
42_glucose 1-phosphate (G1P)	-4.2	-0.1	-0.39	NA	RT-CCS
43_cis,cis-muconic acid	-15	-0.08	-0.07	NA	RT-CCS
46_glutamine	-2.4	-0.12	-0.1	0.976547907	RT-CCS-DIA
47_xylitol	1.1	0.01	-0.17	0.973815598	RT-CCS-DIA
48_trehalose	2	0.05	0.11	0.984183528	RT-CCS-DIA
49_phenylalanine	-12.7	0	0.19	NA	RT-CCS
50_arabitol	1.9	0.01	-0.17	NA	RT-CCS
53_erythrose 4-phosphate (E4P)	-7	0	0.19	NA	RT-CCS
54_sedoheptulose 7-phosphate (S7P)	-5.8	-0.28	-0.26	0.980212087	RT-CCS-DIA
55_tryptophan	-8.3	0.02	0.04	0.966494429	RT-CCS-DIA
56_glutathione reduced	-5.4	-0.22	-0.15	0.986326653	RT-CCS-DIA
57_mannitol	-0.6	-0.04	0.21	0.985697439	RT-CCS-DIA
58_uridine	-2.1	-0.26	0.12	NA	RT-CCS
60_acetyl-CoA	-16.6	0.06	-0.19	0.961652161	RT-CCS-DIA
63_uridine monophosphate	-6	0.05	0.19	0.98602775	RT-CCS-DIA
64_L-threonine	-15.1	0.01	-0.05	0.96017584	RT-CCS-DIA

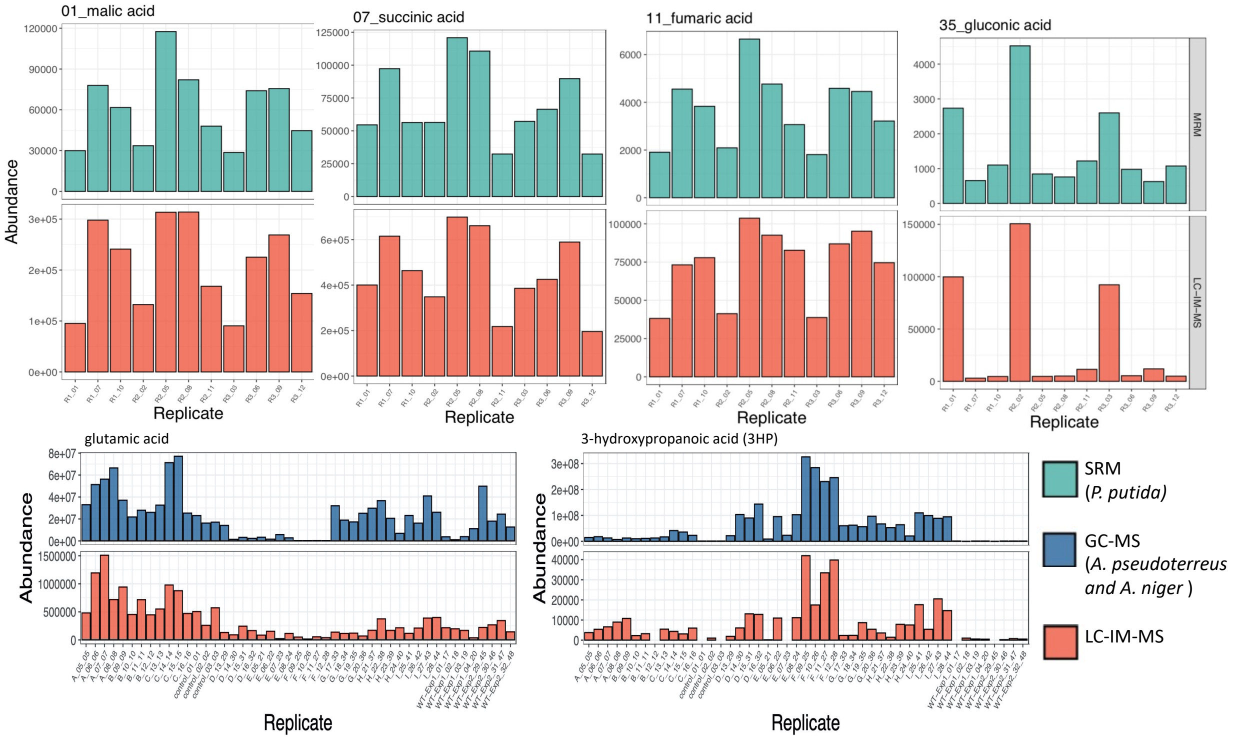

Supplementary Figure 7. Comparison of LC-IM-MS vs SRM and GC-MS relative quantitation results of selected metabolites detected in microbial samples. Similar trends were observed for the metabolites identified in common by the platforms. Source data are provided as a Source Data file.

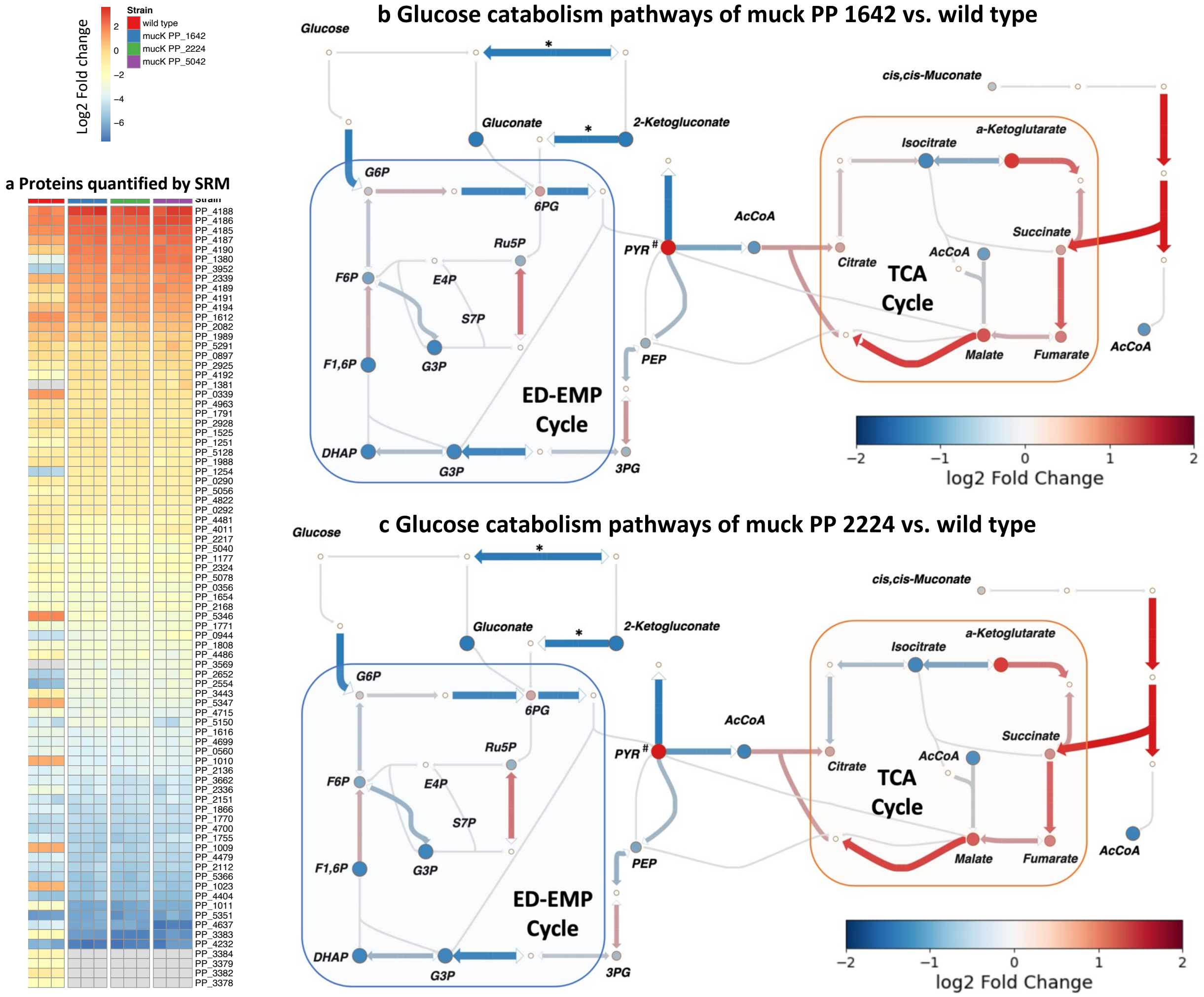

Supplementary Figure 8. Metabolomics and proteomics profiling of *P. putida* wild type and engineered muconate-catabolizing strains. a Global proteomics profiling by SRM. Gray color indicates missing values. **b-c** Glucose and muconate catabolism pathways of mucK PP1642 and PP2224, with fold changes over the wild type. Molecules are represented by circles for LC-IM-MS metabolomics and arrows for SRM proteomics. Symbols indicate protein detection (*: detected in the wild type but not detected in the mucK samples, #: detected in the mucK but not in the wild type. Source data are provided as a Source Data file.

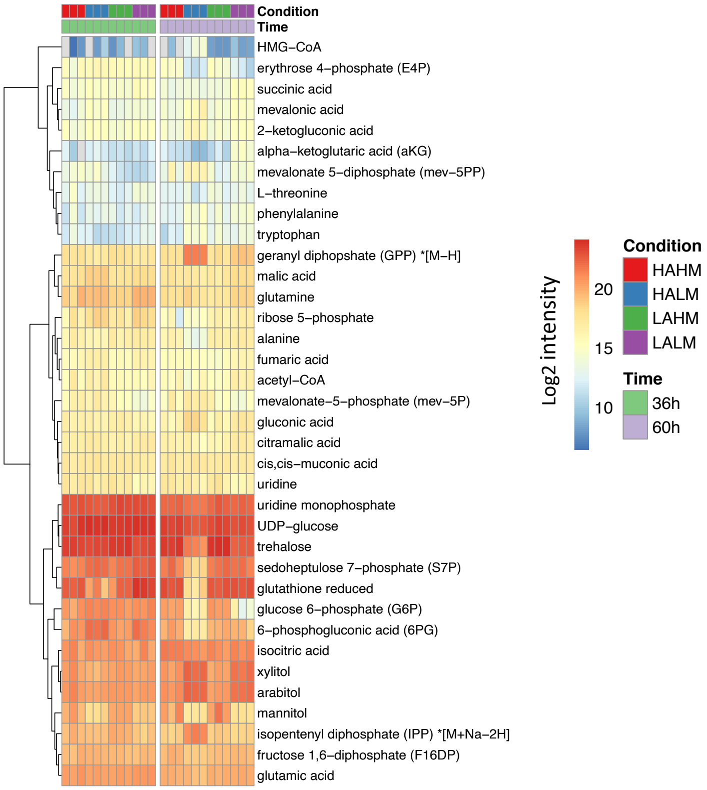

Supplementary Figure 9. Metabolomics profiling of bisabolene producing *R. toruloides* strains. Intracellular metabolites extracted from samples collected at 36 and 60 hr and analyzed using LC-IM-MS. Gray color indicates missing values. Source data are provided as a Source Data file.

Supplementary Table 5. Comparison of the levels of enzyme HMGR and metabolite HMGCoA in *R. toruloides* GB2 grown in HALM (high ash, low moisture) vs all other conditions at 60 hr. The other strains were grown in hydrolysates categorized as HAHM (high ash, high moisture), LAHM (low ash, high moisture) and LALM (low ash, low moisture). A Dunnett test was performed for quantitative difference, which inherently adjusts for the multiple comparisons.

	log2 fold change at 60 hr			Adjusted p-value at 60 hr		
	HALM vs HAHM	HALM vs LAHM	HALM vs LALM	HALM vs HAHM	HALM vs LAHM	HALM vs LALM
HMGR	-1.16	-1.47	-1.23	1.82E-03	5.25E-04	1.39E-04
HMG-CoA	4.66	5.96	5.20	1.39E-03	5.45E-05	1.18E-04
