# Supplementary material for: PeakDecoder enables machine learning-based metabolite annotation and accurate profiling in multidimensional mass spectrometry measurements
Source: Nat Commun. 2023 Apr 28;14:2461. doi: 10.1038/s41467-023-37031-9 (PMC10147702; doi:10.1038/s41467-023-37031-9)
Supplement: Supplementary file 4 — Reporting Summary [file 41467_2023_37031_MOESM4_ESM.pdf]

nature portfolio

Corresponding author(s): Aivett Bilbao and Kristin E. Burnum-Johnson

Last updated by author(s): Feb 12, 2023

Reporting Summary

Nature Portfolio wishes to improve the reproducibility of the work that we publish. This form provides structure for consistency and transparency in reporting. For further information on Nature Portfolio policies, see our Editorial Policies and the Editorial Policy Checklist.

Statistics

For all statistical analyses, confirm that the following items are present in the figure legend, table legend, main text, or Methods section.

n/a	Confirmed	
☐	☒	The exact sample size (*n*) for each experimental group/condition, given as a discrete number and unit of measurement
☐	☒	A statement on whether measurements were taken from distinct samples or whether the same sample was measured repeatedly
☐	☒	The statistical test(s) used AND whether they are one- or two-sided<br>*Only common tests should be described solely by name; describe more complex techniques in the Methods section.*
☒	☐	A description of all covariates tested
☒	☐	A description of any assumptions or corrections, such as tests of normality and adjustment for multiple comparisons
☐	☒	A full description of the statistical parameters including central tendency (e.g. means) or other basic estimates (e.g. regression coefficient) AND variation (e.g. standard deviation) or associated estimates of uncertainty (e.g. confidence intervals)
☐	☒	For null hypothesis testing, the test statistic (e.g. *F*, *t*, *r*) with confidence intervals, effect sizes, degrees of freedom and *P* value noted<br>*Give P values as exact values whenever suitable.*
☒	☐	For Bayesian analysis, information on the choice of priors and Markov chain Monte Carlo settings
☒	☐	For hierarchical and complex designs, identification of the appropriate level for tests and full reporting of outcomes
☒	☐	Estimates of effect sizes (e.g. Cohen's *d*, Pearson's *r*), indicating how they were calculated

Our web collection on statistics for biologists contains articles on many of the points above.

Software and code

Policy information about availability of computer code

Data collection	The MassHunter data acquisition software (v.B.09.00 (B9044.0), Agilent Technologies) was used to collect all ion mobility-mass spectrometry raw data files.
Data analysis	CCS were calculated using the IM-MS Browser (v.10.0, Agilent Technologies). The PNNL-Preprocessor (v2020.07.24) was used to apply moving average smoothing and filtering of the raw data. MS-DIAL (v.4.70) was used to perform untargeted feature finding and MS/MS deconvolution. Skyline (v.64.21.1.0.146) was used to perform targeted data extraction. Implementation of the PeakDecoder algorithm and evaluation of the results were performed in R (v.4.1.0) using packages e1071 (v.1.7-9) and ggplot2 (v.3.3.3).<br><br>The source code of the PeakDecoder algorithm, the library built from standards, and all the input files and results can be found at https://github.com/EMSL-Computing/PeakDecoder. The source code of the automated chromatographic method selection software can be found at https://github.com/poorey/AMSS.<br><br>Statistical analysis of abundances was performed in R using the pmartR package (v0.9.0). Clustered heatmaps were generated using the R package pheatmap (v1.0.12). Bar and error bar plots shown on the metabolic pathway maps were generated using the python package matplotlib (v3.5.1). Chemical structures were drawn using ChemDraw (v19.0). The metabolic pathway maps were visualized using the python packages escher (v1.7.3) and cobrapy (v0.22.1).<br>Skyline (v.64.21.1.0.146) was used for SRM metabolomics data processing.<br>Metabolite Detector (v2.5) was used for GC-MS data processing.<br>Skyline (v64.22.2.1.278) was used for targeted proteomics data processing.<br>MaxQuant (v1.6.2.10) was used for global proteomics database search and statistical analysis of abundances was performed in R using the

pmartR package (v0.9.0).

For manuscripts utilizing custom algorithms or software that are central to the research but not yet described in published literature, software must be made available to editors and reviewers. We strongly encourage code deposition in a community repository (e.g. GitHub). See the Nature Portfolio guidelines for submitting code & software for further information.

Data

Policy information about availability of data

All manuscripts must include a data availability statement. This statement should provide the following information, where applicable:

- Accession codes, unique identifiers, or web links for publicly available datasets
- A description of any restrictions on data availability
- For clinical datasets or third party data, please ensure that the statement adheres to our policy

The microbial LC-IM-MS data (and related Skyline projects) generated in this study have been deposited in the MassIVE database under accession code MSV000089733 [https://doi.org/doi:10.25345/C52R3P17Z]. The P. putida targeted proteomics data generated in this study have been deposited in the Panorama database [https://doi.org/10.6069/6j7y-t592]. The R. toruloides global proteomics data generated in this study have been deposited in the MassIVE database under accession code MSV000091202 [https://doi.org/doi:10.25345/C50K26N04].

Human research participants

Policy information about studies involving human research participants and Sex and Gender in Research.

Reporting on sex and gender	n/a
Population characteristics	n/a
Recruitment	n/a
Ethics oversight	n/a

Note that full information on the approval of the study protocol must also be provided in the manuscript.

Field-specific reporting

Please select the one below that is the best fit for your research. If you are not sure, read the appropriate sections before making your selection.

☒ Life sciences ☐ Behavioural & social sciences ☐ Ecological, evolutionary & environmental sciences

For a reference copy of the document with all sections, see nature.com/documents/nr-reporting-summary-flat.pdf

Life sciences study design

All studies must disclose on these points even when the disclosure is negative.

Sample size	No sample-size calculation was performed. Biological replicates in this study were independent cultures of each microorganism strain of interest. There is low intra-sample variation due to high control over each culture.
Data exclusions	No raw files were excluded from the analyses.
Replication	Each microorganism strain and condition was prepared 3-4 times. Excepting the P. putida muck PP1642 strain which had 2 sample replicates available. All replication attempts were successful and data from each biological replicate is reported in the supplemental tables and figures. All molecules of interest discussed in the manuscript were statistically significant (<0.05 p-value).
Randomization	Each microorganism (wildtype and mutant strains) was treated as an experimental group were samples were processed and analyzed using randomized orders.
Blinding	Investigators were not blinded to group allocation during data collection or analysis. Knowledge of group assignment did not affect analysis results since conclusions were based on confident molecular identification metrics and statistics.

Reporting for specific materials, systems and methods

We require information from authors about some types of materials, experimental systems and methods used in many studies. Here, indicate whether each material, system or method listed is relevant to your study. If you are not sure if a list item applies to your research, read the appropriate section before selecting a response.

Materials & experimental systems

n/a	Involved in the study
[x]	[ ] Antibodies
[x]	[ ] Eukaryotic cell lines
[x]	[ ] Palaeontology and archaeology
[ ]	[x] Animals and other organisms
[x]	[ ] Clinical data
[x]	[ ] Dual use research of concern

Methods

n/a	Involved in the study
[x]	[ ] ChIP-seq
[x]	[ ] Flow cytometry
[x]	[ ] MRI-based neuroimaging

Animals and other research organisms

Policy information about studies involving animals; ARRIVE guidelines recommended for reporting animal research, and Sex and Gender in Research

Laboratory animals	Aspergillus pseudoterreus, Aspergillus niger, Pseudomonas putida and Rhodosporidium toruloides strains.
Wild animals	The study did not involve wild animals.
Reporting on sex	n/a
Field-collected samples	The study did not involve samples collected from the field.
Ethics oversight	No ethical approval or guidance was required.

Note that full information on the approval of the study protocol must also be provided in the manuscript.
